# Effectiveness of the 23-Valent Pneumococcal Polysaccharide Vaccine Among Elderly Adults in Changshu City: A Multilevel Cluster Analysis

**DOI:** 10.3390/vaccines14030223

**Published:** 2026-02-28

**Authors:** Wancheng Zhang, Wenting He, Zhengyuan Zhou, Tingting Xu, Xin Li, Ning Zhang

**Affiliations:** 1 Changshu Center for Disease Prevention and Control, Changshu 215500, China; fairyfay@163.com (W.Z.); cscdczzy@163.com (Z.Z.); 2 School of Public Health, Nanjing Medical University, Nanjing 211166, China; hewenting@stu.njmu.edu.cn (W.H.); xutingting@stu.njmu.edu.cn (T.X.)

**Keywords:** pneumococcal pneumonia, elderly adults, PPV23, vaccine effectiveness, risk factors, multilevel cluster study

## Abstract

Background: Elderly individuals are at high risk for pneumococcal pneumonia, and vaccination is crucial for prevention. This study aims to evaluate the effectiveness of the 23-valent pneumococcal polysaccharide vaccine (PPV23) in elderly individuals aged 65–84 years in Changshu City, identify key target groups, and explore potential factors influencing vaccine effectiveness. Methods: A multilevel cluster sampling method was employed to survey 2310 elderly individuals aged 65–84 years from four randomly selected towns/streets in Changshu City. Data were collected through face-to-face questionnaires and integrated with the immunization records system and electronic health records. Chi-square tests and multivariable logistic regression models were used to assess the association between PPV23 vaccination and pneumonia risk, with adjustments for potential confounding factors. Results: PPV23 vaccination significantly reduced the risk of pneumonia (adjusted OR = 0.69, 95% CI = 0.50–0.94, *p* = 0.019). The protective effect was more pronounced in individuals aged 75–79 years (OR = 0.56, 95% CI = 0.32–0.95) and females (OR = 0.48, 95% CI = 0.28–0.79). Chronic respiratory disease, survey location, gender, and age were identified as independent factors influencing vaccine effectiveness. Conclusions: PPV23 vaccination is effective in reducing pneumonia risk among elderly adults, particularly in specific subgroups. Future vaccination strategies should prioritize targeted programs for high-risk groups to improve coverage and effectiveness.

## 1. Introduction

Streptococcus pneumoniae, a Gram-positive bacterium, is a leading cause of infectious diseases, including community-acquired pneumonia (CAP) and invasive pneumococcal diseases (IPDs) such as sepsis and meningitis [1,2]. It is a major contributor to lower respiratory tract infections and related mortality, particularly among high-risk groups such as children, the elderly, and immunocompromised individuals [3,4]. Pneumococcal pneumonia not only poses a significant threat to individual health but also imposes a substantial social and economic burden, especially in the elderly population, where it is a leading cause of morbidity and mortality [5]. According to the World Health Organization (WHO), pneumococcal diseases are responsible for approximately 1.6 million deaths annually, with disproportionately high incidence and mortality rates among the elderly population [6]. Therefore, the development and promotion of effective pneumococcal vaccines are critical for public health.

In China, the rapid aging of the population has heightened the vulnerability of the elderly to infectious diseases, with pneumonia being a particularly pressing concern. The incidence of CAP in the elderly is more than ten times higher than in younger individuals, and it increases progressively with age. Notably, individuals over 60 years old account for 81.2% of all pneumonia cases [7]. For this high-risk group, vaccination with the 23-valent pneumococcal polysaccharide vaccine (PPV23) can effectively prevent pneumococcal infections [8]. First introduced in the United States in 1983, PPV23 was officially approved for use in China in 1996 [9]. Numerous studies, both in China and globally, have demonstrated the efficacy of PPV23 in preventing pneumococcal pneumonia among the elderly [10,11]. In both immunocompetent adults and elderly populations, PPV23 vaccination significantly increases the levels of serotype-specific anticapsular antibodies, providing robust immune protection [8,12,13,14,15,16,17]. While PPV23 has been widely studied, data on its effectiveness in specific regions like Changshu City remain limited.

Immunization programs are a cornerstone of public health prevention and control. Vaccination, as a cost-effective and efficient intervention, remains the most impactful public health strategy for disease prevention. In Changshu City, the local government has implemented a proactive immunization program as part of the Suzhou Healthy City “531” initiative, launched in 2018 to provide free PPV23 to residents aged 65 years and older. Between 2018 and 2022, approximately 80,000 elderly individuals received the vaccine. This study aims to evaluate the effectiveness of PPV23 vaccination in the elderly population of Changshu City and identify potential factors influencing its efficacy. The findings will provide scientific evidence to inform government agencies in developing targeted immunization strategies for pneumonia prevention in the elderly.

## 2. Materials and Methods

### 2.1. Study Design and Population

This study was conducted in Changshu City from October 2021 to November 2022, using a multilevel cluster sampling approach. The study population consisted of elderly individuals aged 65 to 84 years who were registered residents of Changshu City. Four towns/streets were randomly selected from Changshu City, and with each town/street, one township health center (or community health service center) was randomly chosen. Elderly individuals aged 65 to 84 years were recruited from these centers. The inclusion criteria were: (1) registered residents of Changshu City aged 65–84 years; (2) willingness to participate in the study. The exclusion criteria were: (1) individuals with cognitive or intellectual impairments that hindered effective communication; (2) incomplete questionnaire responses; (3) refusal to participate in the survey. Based on the records from the Jiangsu Provincial Immunization Comprehensive Service Management Information System, participants were categorized into the vaccination group (those who received PPV23) and the control group (those who did not receive PPV23). The two groups were matched 1:1 by age and gender.

### 2.2. Data Collection

Data were collected through a combination of centralized surveys (conducted alongside routine elderly health examinations) and household surveys. Trained survey personnel administered face-to-face questionnaires, while additional information (e.g., medical history, insurance status) was obtained from the city’s electronic health records and health insurance database. Prior to the formal survey, a pilot study was conducted to refine and optimize the questionnaire. The final questionnaire comprised four sections: (1) Socio-demographic characteristics: age, gender, living arrangements, education level, pre-retirement occupation, average monthly income in the past year, medical insurance status, and self-reported health status; (2) Disease and vaccine awareness: medical history, knowledge, attitudes, and practices related to pneumonia and vaccines, as well as influenza vaccination history; (3) Lifestyle and behavioral factors: daily handwashing frequency, ventilation habits in living and workplaces, and current smoking status; (4) Disease burden: occurrence of pneumonia-related illnesses in the past year.

### 2.3. Ethics Statement

All participants provided written informed consent prior to participation. The study protocol was reviewed and approved by the Ethics Committee of the Changshu City Center for Disease Prevention and Control.

### 2.4. Statistical Analysis

Survey data were double-entered using Epidata software (version 3.1), and the baseline database was verified by dedicated personnel. (1) Descriptive analysis: socio-demographic characteristics were summarized using frequencies and percentages; (2) Vaccine effectiveness: initial assessment of PPV23 effectiveness was performed using the chi-square (χ^2^) test; (3) Stratified analysis: to identify key target populations for vaccination, stratified analyses were conducted. Logistic regression models were used to adjust for potential confounding factors. For strata with small sample sizes or complete separation issues, Firth’s logistic regression was applied to ensure robust estimates; (4) Factors influencing vaccine effectiveness: univariate and multivariate logistic regression analyses were performed. Variables with *p*-values < 0.05 in the χ^2^ test were included as independent variables in the multivariate logistic regression model to evaluate their independent effects on vaccine effectiveness. All analyses were conducted using R software (version 4.4.2) with two-sided *p*-values < 0.05 considered statistically significant.

## 3. Results

### 3.1. Characteristics of the Study Population

A total of 2402 questionnaires were collected, with 2310 valid responses, yielding a validity rate of 96.17%. By 31 December 2023, medical information for 2212 participants was successfully matched with electronic health records and insurance data of Changshu city registered residents. The remaining 98 participants were contacted via a household survey to obtain their health information. Among the 2310 participants, 933 had received the PPV23 vaccination, while 1377 were unvaccinated, resulting in a vaccination coverage rate of 40.39%.

The participants were aged between 65 and 84 years, with a mean age of 73.02 ± 4.92 years. The male-to-female ratio was 1:1.23. The majority of the elderly participants had a low education level, with 88.83% having completed primary school or below. More than half (53.42%) lived with their children, and 63.20% were former farmers. Over half (55.11%) had an average monthly income of less than 1000 yuan. Nearly all participants had health insurance, with 91.39% reporting coverage. PPV23 vaccination dates ranged from 2018 to 2022, and the survey was conducted from 2021 to 2022.

### 3.2. Association Between PPV23 Vaccination and Risk of Pneumonia

Among the 2310 participants, 199 developed pneumonia (8.61%), with 66 cases in the vaccination group and 133 cases in the control group. The remaining 2111 participants did not develop pneumonia (91.39%). χ^2^ test results indicated that PPV23 vaccination was a significant protective factor against pneumonia occurrence (OR = 0.71, 95% CI = 0.52–0.97, *p* = 0.030) (Table 1). This finding suggested that the PPV23 vaccination effectively reduced the risk of pneumonia, with a 29% lower risk in the vaccination group compared to the control group.

### 3.3. Stratified Analysis of PPV23 Vaccination and Risk of Pneumonia

To further elucidate the effectiveness of PPV23 vaccination, stratified analyses were conducted, adjusting for potential confounding factors such as age and gender. The overall adjusted analysis demonstrated that PPV23 vaccination continued to exhibit a protective effect against pneumonia (OR = 0.69, 95% CI = 0.50–0.94, *p* = 0.019), corresponding to a 31% risk reduction in pneumonia risk.

Next, stratified analysis was performed to assess the impact of PPV23 vaccination on pneumonia risk across socio-demographic characteristics. The most significant protective effects were observed in individuals aged 75–79 years (OR = 0.56, 95% CI = 0.32–0.95), females (OR = 0.48, 95% CI = 0.28–0.79), those with primary school education or below (OR = 0.70, 95% CI = 0.50–0.97), those living with children (OR = 0.60, 95% CI = 0.40–0.91), those with an average monthly income of less than 1000 CNY (OR = 0.50, 95% CI = 0.33–0.74), and those with medical insurance (OR = 0.70, 95% CI = 0.50–0.96).

Further stratification by disease status and vaccine awareness revealed that the protective effects were more pronounced in individuals without chronic respiratory disease (OR = 0.63, 95% CI = 0.45–0.87), those aware of pneumonia (OR = 0.55, 95% CI = 0.35–0.84), those not worried about contracting pneumonia (OR = 0.62, 95% CI = 0.39–0.97), those who believed pneumonia is preventable (OR = 0.65, 95% CI = 0.43–0.99), those who had heard of the pneumococcal vaccine (OR = 0.59, 95% CI = 0.36–0.95), those aware of the free pneumonia vaccination program for the elderly (OR = 0.54, 95% CI = 0.31–0.93), and those who had not received the influenza vaccine (OR = 0.69, 95% CI = 0.50–0.94).

Similarity, analysis of lifestyle and behavioral factors showed that the protective effects were more evident in individuals living in rural dispersed areas (OR = 0.59, 95% CI = 0.39–0.86), those living or working in environments without air purifiers or ventilation systems (OR = 0.69, 95% CI = 0.50–0.95), those who opened windows for ventilation in living or workspaces (OR = 0.68, 95% CI = 0.49–0.94), those who washed their hands more than eight times daily (OR = 0.57, 95% CI = 0.34–0.92), those with the habit of washing hands before meals (OR = 0.66, 95% CI = 0.47–0.91), those who washed hands after returning home from outings (OR = 0.60, 95% CI = 0.42–0.84), non-smokers (OR = 0.65, 95% CI = 0.45–0.92), and those living with household members who smoke (OR = 0.36, 95% CI = 0.19–0.63) (Table 2).

### 3.4. Univariate Analysis of Factors Affecting PPV23 Vaccination Effectiveness

To identify factors associated with the effectiveness of PPV23 vaccination, we carried out univariate analyses. The results revealed significant differences in survey location (χ^2^ = 15.80, *p* = 0.001), age (W = 22,215, *p* = 0.002), gender (χ^2^ = 14.26, *p* < 0.001), and chronic respiratory disease (χ^2^ = 25.65, *p* < 0.001) between pneumonia and non-pneumonia groups (Table 3).

### 3.5. Multivariate Logistic Regression Analysis of Factors Affecting PPV23 Vaccination Effectiveness

To identify the independent factors influencing the effectiveness of the PPV23 vaccine, variables with *p* < 0.05 in the univariate analysis were included in a multivariate logistic regression model, with the effectiveness of the PPV23 vaccine as the dependent variable (pneumonia = 1, non-pneumonia = 0). The regression results showed that survey location, gender, age, and chronic respiratory disease were independent factors affecting PPV23 vaccination effectiveness, with age being a risk factor for pneumonia.

Notably, age emerged as a risk factor for pneumonia, with each additional year of age associated with a 7% increase in pneumonia risk (OR = 1.07, 95% CI = 1.01–1.14, *p* = 0.020). In terms of survey locations, the risk of pneumonia was significantly lower in Zhitang Town (OR = 0.33, 95% CI = 0.17–0.62, *p* = 0.001) and Shanghu Town (OR = 0.28, 95% CI = 0.11–0.62, *p* = 0.003) compared to Shajiabang Town. However, the difference was not statistically significant for Changfu Street (OR = 0.65, 95% CI = 0.27–1.41, *p* = 0.293), suggesting an inconclusive effect. Regarding gender, females exhibited a significantly lower risk of pneumonia than males (OR = 0.42, 95% CI = 0.24–0.73, *p* = 0.002). Furthermore, the absence of chronic respiratory disease was identified as a protective factor, with the risk of pneumonia being only 0.16 times that of individuals with chronic respiratory disease (OR = 0.16, 95% CI = 0.07–0.35, *p* < 0.001). These findings underscore that chronic respiratory disease had the most substantial impact on PPV23 vaccine effectiveness, followed by survey location, gender, and age (Table 4).

## 4. Discussion

This study demonstrated that PPV23 vaccination significantly reduces the risk of pneumonia in the elderly, consistent with previous findings [18,19,20]. Before adjusting for confounding factors, vaccination reduced pneumonia incidence by 28.8% in the target population. After adjustment, the OR value for vaccination was 0.69, indicating a 31% reduction in pneumonia risk. This highlights the robust protective effect of PPV23 vaccination and underscores its value as a public health policy.

The PPV23 vaccination rate among the elderly population aged 65–84 years in Changshu City was 40.39%, which is moderate compared to national levels [21,22,23,24] but far below rates in developed countries like the United States [25]. Identifying high-benefit populations and key factors influencing vaccine effectiveness is crucial for targeted vaccination strategies.

Our stratified analysis showed that PPV23 vaccination provided the greatest benefits in individuals aged 75–79 years, females, those with low education levels, low income, medical insurance, no chronic respiratory disease, awareness of pneumonia, belief in its preventability, knowledge of pneumococcal vaccines and free vaccination programs, and those not receiving the influenza vaccine. Behavioral factors such as frequent handwashing, good ventilation practices, and non-smoking also enhanced vaccine effectiveness. Notably, living in rural dispersed areas or with smokers further increased the protective effect. These groups experienced a pneumonia incidence reduction of 30–64%. Government authorities should prioritize these specific subgroups in their vaccination policies and interventions to maximize public health benefits.

Smoking behavior is a key factor influencing pneumonia risk, highlighting the need for integrated smoking cessation interventions. Smoking is a well-established major risk factor for pneumonia, with a clear dose–response relationship between smoking exposure and pneumonia incidence [26]. Importantly, individuals who quit smoking for at least 10 years see their pneumonia risk significantly reduced to levels comparable to non-smokers [27]. Additionally, household secondhand smoke exposure is a significant contributor to respiratory disease risk. Passive smoking, especially among individuals aged ≥65 years, significantly increases pneumonia risk [28]. A meta-analysis reported that passive smoking in this age group increases CAP risk by approximately 64% [29]. Tobacco smoke can substantially elevate oxidative stress, particularly in the lungs. Prolonged or repeated exposure can damage respiratory epithelial cells, pulmonary connective tissue, and vascular endothelium, compromising local barrier integrity and defense mechanisms. As a result, these tissues become more susceptible to infections and inflammatory insults. Importantly, such adverse effects may occur even at low levels of smoke exposure [30,31,32,33]. Considering these mechanisms, exposure to secondhand smoke may significantly elevate the risk of community-acquired pneumonia [34,35]. These findings underscore the importance of considering both active and passive smoking when evaluating risk factors for pneumonia. Given these findings, it is imperative to intensify public health campaigns promoting smoking cessation and awareness of smoking’s harmful effects on respiratory health. Increasing PPV23 vaccination coverage among the elderly could also provide additional protection and enhance public health outcomes.

Our study identified chronic respiratory disease as the most important factor influencing PPV23 vaccine effectiveness. Individuals without chronic respiratory disease had a significantly lower risk of pneumonia, indicating higher susceptibility among those with such conditions. A prospective cohort study by Satoshi Inoue et al. [36] confirmed that PPV23 vaccine effectiveness was significantly influenced by prior pulmonary infection history. Specifically, patients with chronic respiratory failure but no prior lung infections had a significantly reduced risk of pulmonary infections post-vaccination. These findings highlight the importance of early PPV23 vaccination in elderly patients with chronic lung diseases to maximize preventive benefits.

Survey location was the second most significant factor influencing PPV23 vaccine effectiveness. In our analysis, pneumonia incidence in PPV23-vaccinated individuals was significantly lower at Zhitang Town (4.2%) and Shanghu Town (5.1%) compared to the other two sites (12.7% and 7.4%), confirmed by multivariate logistic regression. This may be due to higher vaccination rates in these areas (66.05% and 37.04% vs. 34.03% and 22.93% in other sites) [37]. These differences likely reflect local governments’ active promotion of vaccination campaigns and free vaccination policies, which enhanced elderly participation and strengthened PPV23’s protective effects.

Gender significantly influenced the effectiveness of the PPV23 vaccine, with females showing a lower risk of pneumonia than males. This aligns with findings by Klein SL et al. [38], who noted that females, especially elderly females, are more likely to experience and report adverse reactions after vaccination. Timothy L. Wiemken et al. [39] showed that while PPV23 reduced the risk of hospitalization due to pneumococcal infections in the elderly, this effect was more pronounced in females, likely due to gender differences in immune responses. Consistent with this, other studies [38,40] have highlighted that females typically mount stronger antibody responses and exhibit different immune regulatory mechanisms compared to males, indicating better immune protection against infections [41].

In our study, age was an independent risk factor for PPV23 vaccine effectiveness, with pneumonia risk increasing significantly with age. This aligns with findings by Werner C. Albrich et al. [42] and Hayato Yamana et al. [20], who showed that PPV23 reduced pneumonia-related hospitalization in individuals ≥65 years, but its effectiveness diminished with increasing age, especially in those ≥90 years. This decline is attributed to immunosenescence, characterized by weakened T-cell and B-cell functions, which impairs pathogen response and vaccine efficacy [43,44,45]. Therefore, tailored vaccination strategies for different age groups are needed, especially for the elderly. Future efforts should focus on exploring more effective vaccines, optimizing vaccination methods, and improving vaccination coverage to reduce pneumonia incidence and enhance the health and quality of life of the elderly.

We found that PPV23 showed a significant protective effect against pneumonia in older adults living with children. Information on whether households included young children was not specifically collected, so their presence could not be directly assessed. It is therefore likely that some older adults living with their children also co-reside with young household members. Adult children typically provide care for elderly parents; when young children are present, older adults inevitably have contact with them and may participate in their care. Studies have shown that young children play an important role in respiratory virus transmission among adults, particularly in cases of CAP and in patients with chronic obstructive pulmonary disease (COPD) [46,47,48,49]. Future studies are needed to further investigate the potential role of young children in pneumococcal transmission within households.

In this study, the interval between vaccination and the survey among participants ranged from less than 1 year to 4 years. Previous studies targeting adults aged ≥65 years have shown that the protective effect of PPV23 is relatively high within the first 2 years after vaccination (approximately 40%), decreases to around 30% during the 2–4 year period, and declines further after 5 years, indicating that vaccine effectiveness may gradually wane over time [50]. Considering that the participants in this study were within 1–4 years post-vaccination, the PPV23 continues to confer a moderate degree of protection during this interval, but differences in time since vaccination may have some impact on the estimated effect and should therefore be interpreted with caution.

According to the U.S. CDC’s Advisory Committee on Immunization Practices (ACIP), individuals aged 65 years and older who have previously received a dose of PPV23 should receive a revaccination five years after their initial dose [51]. PPV23 has been shown to provide approximately 75% effectiveness against invasive disease after the initial dose, with protection maintained at roughly 74% after revaccination [52].

Our study has limitations. Accurate data on medical expenses and indirect costs were unavailable due to challenges in data collection and elderly individuals’ memory issues. This limits our ability to conduct a comprehensive health economic evaluation. Future studies should focus on increasing vaccination rates in high-risk groups and exploring more effective vaccines or vaccination methods. Additionally, caution is needed when interpreting stratified analyses with small sample sizes or complete separation, and future research should aim to validate these findings with larger samples.

## 5. Conclusions

This study shows that the PPV23 vaccine significantly reduces pneumonia risk, especially in elderly subgroups aged 75–79 years, females, those with low education, low income, and no chronic respiratory disease. Behavioral factors such as frequent handwashing, good ventilation, and non-smoking also enhance vaccine protection. Notably, living in rural areas or with smokers further increases vaccine effectiveness. Chronic respiratory disease, location, gender, and age are the main factors influencing vaccine effectiveness.

Future strategies should prioritize these subgroups to optimize vaccine coverage and effectiveness. Public health efforts should focus on increasing pneumonia prevention awareness, promoting vaccination, and integrating smoking cessation interventions, especially among high-risk populations with chronic respiratory conditions.

## Figures and Tables

**Table 1:** The effect of PPV23 vaccination on the risk of pneumonia.

Groups	No. of Participants	Pneumonia (*n* = 199)		Non-Pneumonia (*n* = 2111)		*p*-Value ^a^	OR (95% CI) ^a^
		*n*	%	*n*	%		
Vaccination	933	66	7.1	867	92.9	**0.030**	**0.71 (0.52–0.97)**
Control	1377	133	9.7	1244	90.3		

Bold elements are used to highlight positive outcomes. In the *p*-values, bold indicates *p*-values < 0.05, with the specific *p*-values provided. In the OR values, bold is used solely to highlight protective effects and does not indicate their significance (Same as the following tables). ^a^ two-sided χ^2^ test.

**Table 2:** Evaluation of PPV23 vaccination effectiveness in study participants.

Variables	No. of Pneumonia/No. of Non-Pneumonia	Vaccination Group		Control Group		*p*-Value ^a^	OR (95% CI) ^a^
		*n*	%	*n*	%		
Total	199/2111	66/867	33.2/41.1	133/1244	66.8/58.9	0.019	0.69 (0.50–0.94)
Survey locations							
Shajiabang Town	107/563	29/199	27.1/35.3	78/364	72.9/64.7	0.107	0.68 (0.42–1.08)
Changfu Street	45/487	9/113	20.0/23.2	36/374	80.0/76.8	0.656	0.84 (0.37–1.74)
Zhitang Town	33/562	20/373	60.6/66.4	13/189	39.4/33.6	0.524	0.79 (0.39–1.67)
Shanghu Town	14/499	8/182	57.1/36.5	6/317	42.9/63.5	0.143	2.24 (0.76–6.92)
Age (years)							
65~69	37/617	9/192	24.3/31.1	28/425	75.7/68.9	0.480 ^c^	0.76 (0.33–1.58) ^c^
70~74	51/750	23/372	45.1/49.6	28/378	54.9/50.4	0.555 ^c^	0.84 (0.47–1.49) ^c^
75~79	69/507	22/226	31.9/44.6	47/281	68.1/55.4	0.035 ^c^	0.56 (0.32–0.95) ^c^
80~84	42/237	12/77	28.6/32.5	30/160	71.4/67.5	0.533 ^c^	0.79 (0.37–1.61) ^c^
Gender							
Male	114/921	45/376	39.5/40.8	69/545	60.5/59.2	0.535 ^d^	0.88 (0.59–1.31) ^d^
Female	85/1190	21/491	24.7/41.3	64/699	75.3/58.7	0.005 ^d^	0.48 (0.28–0.79) ^d^
Education level							
Primary school and below	175/1877	58/771	33.1/41.1	117/1106	66.9/58.9	0.033	0.70 (0.50–0.97)
Middle school	21/202	8/88	38.1/43.6	13/114	61.9/56.4	0.454	0.70 (0.26–1.76)
High school	3/29	0/7	0.0/24.1	3/22	100.0/75.9	0.566 ^b^	0.43 (0.003–5.81) ^b^
Associate degree and above	0/3	0/1	0.0/33.3	0/2	0.0/66.7	- ^b^	- ^b^
Living status							
Living alone	35/261	12/108	34.3/41.4	23/153	65.7/58.6	0.545	0.79 (0.36–1.67)
Living with spouse	53/727	18/264	34.0/36.3	35/463	66.0/63.7	0.430	0.78 (0.42–1.41)
Living with children	111/1123	36/495	32.4/44.1	75/628	67.6/55.9	0.018	0.60 (0.40–0.91)
Occupation before retirement							
Teacher	1/6	0/3	0.0/50.0	1/3	100.0/50.0	- ^b^	- ^b^
Healthcare worker	1/10	0/3	0.0/30.0	1/7	100.0/70.0	0.951 ^b^	0.90 (0.01–53.25) ^b^
Government employee	2/5	0/1	0.0/20.0	2/4	100.0/80.0	- ^b^	- ^b^
Corporate employee	5/53	3/15	60.0/28.3	2/38	40.0/71.7	0.178	3.91 (0.55–35.43)
Self-employed business owner	6/70	1/30	16.7/42.9	5/40	83.3/57.1	0.207	0.24 (0.01–1.63)
Restaurant/Commercial service worker	2/28	1/8	50.0/28.6	1/20	50.0/71.4	0.803 ^b^	0.71 (0.00–11.91) ^b^
Worker/Migrant worker	33/421	8/135	24.2/32.1	25/286	75.8/67.9	0.218	0.59 (0.24–1.31)
Farmer	137/1323	50/598	36.5/45.2	87/725	63.5/54.8	0.063	0.71 (0.49–1.01)
No fixed occupation	12/195	3/74	25.0/37.9	9/121	75.0/62.1	0.330	0.51 (0.11–1.80)
Average monthly income (CNY)							
<1000	122/1151	37/538	30.3/46.7	85/613	69.7/53.3	<0.001	0.50 (0.33–0.74)
1000~2999	60/795	25/296	41.7/37.2	35/499	58.3/62.8	0.613	1.15 (0.66–1.96)
3000~4999	17/131	4/25	23.5/19.1	13/106	76.5/80.9	0.511	1.52 (0.39–4.99)
≥5000	0/34	0/8	0.0/23.5	0/26	0.0/76.5	- ^b^	- ^b^
Medical insurance							
Yes	186/1925	59/758	31.7/39.4	127/1167	68.3/60.6	0.031	0.70 (0.50–0.96)
No	13/186	7/109	53.8/58.6	6/77	46.2/41.4	0.657	0.77 (0.24–2.54)
Overall health status							
Excellent	3/32	1/15	33.3/46.9	2/17	66.7/53.1	- ^b^	- ^b^
Very good	44/511	13/195	29.5/38.2	31/316	70.5/61.8	0.191	0.64 (0.31–1.23)
Good	77/748	27/296	35.1/39.6	50/452	64.9/60.4	0.310	0.77 (0.47–1.26)
Fair	61/680	21/321	34.4/47.2	40/359	65.6/52.8	0.063	0.59 (0.33–1.02)
Poor	13/126	4/34	30.8/27.0	9/92	69.2/73.0	0.675	1.31 (0.33–4.43)
Don’t know	1/14	0/6	0.0/42.9	1/8	100.0/57.1	- ^b^	- ^b^
Chronic disease							
Yes	143/1355	49/552	34.3/40.7	94/803	65.7/59.3	0.116	0.75 (0.51–1.07)
No	56/756	17/315	30.4/41.7	39/441	69.6/58.3	0.057	0.56 (0.30–1.00)
Chronic respiratory disease							
Yes	26/86	12/35	46.2/40.7	14/51	53.8/59.3	0.445	1.44 (0.56–3.73)
No	173/2025	54/832	31.2/41.1	119/1193	68.8/58.9	0.007	0.63 (0.45–0.87)
Awareness of pneumonia							
Know	108/1106	33/477	30.6/43.1	75/629	69.4/56.9	0.007	0.55 (0.35–0.84)
Don’t Know	91/1005	33/390	36.3/38.8	58/615	63.7/61.2	0.599	0.89 (0.56–1.38)
Do you consider pneumonia a serious disease?							
Yes	78/764	27/312	34.6/40.8	51/452	65.4/59.2	0.289	0.77 (0.46–1.24)
No	31/297	9/107	29.0/36.0	22/190	71.0/64.0	0.457	0.73 (0.30–1.64)
Unclear	90/1050	30/448	33.3/42.7	60/602	66.7/57.3	0.074	0.66 (0.41–1.03)
Are you worried about getting pneumonia?							
Very worried	11/103	3/39	27.3/37.9	8/64	72.7/62.1	0.447	0.58 (0.12–2.17)
Somewhat worried	37/370	14/177	37.8/47.8	23/193	62.2/52.2	0.325	0.70 (0.34–1.41)
Not worried	105/906	29/333	27.6/36.8	76/573	72.4/63.2	0.042	0.62 (0.39–0.97)
Haven’t thought about it	46/732	20/318	43.5/43.4	26/414	56.5/56.6	0.871	0.95 (0.51–1.74)
Do you think pneumonia can be prevented?							
Can be prevented	110/1048	38/459	34.5/43.8	72/589	65.5/56.2	0.046	0.65 (0.43–0.99)
Cannot be prevented	3/31	1/8	33.3/25.8	2/23	66.7/74.2	0.814	1.36 (0.06–16.87)
Unclear	86/1032	27/400	31.4/38.8	59/632	68.6/61.2	0.152	0.71 (0.43–1.13)
Have you heard of the pneumonia vaccine?							
Yes	78/789	30/406	38.5/51.5	48/383	61.5/48.5	0.033	0.59 (0.36–0.95)
No	121/1322	36/461	29.8/34.9	85/861	70.2/65.1	0.177	0.75 (0.49–1.13)
Do you know about the free pneumonia vaccine for the elderly?							
Know	63/678	21/336	33.3/49.6	42/342	66.7/50.4	0.030	0.54 (0.31–0.93)
Don’t know	136/1433	45/531	33.1/37.1	91/902	66.9/62.9	0.207	0.78 (0.53–1.14)
Would you be willing to receive the free pneumonia vaccine?							
Willing	87/1002	36/510	41.4/50.9	51/492	58.6/49.1	0.088	0.68 (0.43–1.06)
Unwilling	46/472	6/109	13.0/23.1	40/363	87.0/76.9	0.114	0.49 (0.18–1.11)
Undecided/Unclear	66/637	24/248	36.4/38.9	42/389	63.6/61.1	0.455	0.82 (0.47–1.38)
Have you ever received the influenza vaccine?							
Yes	4/52	2/27	50.0/51.9	2/25	50.0/48.1	0.994	0.99 (0.10–9.95)
No	195/2059	64/840	32.8/40.8	131/1219	67.2/59.2	0.019	0.69 (0.50–0.94)
Household location							
Urban center	1/23	0/8	0.0/34.8	1/15	100.0/65.2	- ^b^	- ^b^
Suburban area	10/94	3/27	30.0/28.7	7/67	70.0/71.3	0.956	1.04 (0.21–4.26)
Rural town	66/582	22/188	33.3/32.3	44/394	66.7/67.7	0.946	1.02 (0.58–1.75)
Rural dispersed	122/1412	41/644	33.6/45.6	81/768	66.4/54.4	0.008	0.59 (0.39–0.86)
Do you use air purifiers or ventilation systems at home or work?							
Yes	8/104	1/31	12.5/29.8	7/73	87.5/70.2	0.540	0.50 (0.03–3.37)
No	191/2007	65/836	34.0/41.7	126/1171	66.0/58.3	0.023	0.69 (0.50–0.95)
Do you open windows for ventilation at home or work?							
Yes	184/2008	61/830	33.2/41.3	123/1178	66.8/58.7	0.021	0.68 (0.49–0.94)
No	15/103	5/37	33.3/35.9	10/66	66.7/64.1	0.727	0.81 (0.23–2.53)
Hand washing frequency (times per day)							
0	2/13	1/6	50.0/46.2	1/7	50.0/53.8	0.880	1.30 (0.03–50.89)
1~3	29/333	17/166	58.6/49.8	12/167	41.4/50.2	0.316	1.49 (0.69–3.33)
4~8	75/873	24/368	32.0/42.2	51/505	68.0/57.8	0.087	0.64 (0.38–1.06)
>8	93/892	24/327	25.8/36.7	69/565	74.2/63.3	0.024	0.57 (0.34–0.92)
Hand washing frequency with soap or hand sanitizer (times per day)							
0	30/357	13/151	43.3/42.3	17/206	56.7/57.7	0.811	1.10 (0.50–2.34)
1~3	91/966	33/420	36.3/43.5	58/546	63.7/56.5	0.157	0.72 (0.46–1.13)
4~8	53/529	15/218	28.3/41.2	38/311	71.7/58.8	0.054	0.54 (0.28–1.00)
>8	25/259	5/78	20.0/30.1	20/181	80.0/69.9	0.160	0.47 (0.14–1.26)
Do you have a habit of washing hands before meals?							
Yes	184/1953	60/813	32.6/41.6	124/1140	67.4/58.4	0.012	0.66 (0.47–0.91)
No	15/158	6/54	40.0/34.2	9/104	60.0/65.8	- ^b^	- ^b^
Do you have a habit of washing hands after returning home from outings?							
Yes	170/1716	53/723	31.2/42.1	117/993	68.8/57.9	0.004	0.60 (0.42–0.84)
No	29/395	13/144	44.8/36.5	16/251	55.2/63.5	0.451	1.35 (0.61–2.91)
Do you currently smoke?							
Yes	51/448	19/175	37.3/39.1	32/273	62.7/60.9	0.474	0.80 (0.43–1.46)
No	148/1663	47/692	31.8/41.6	101/971	68.2/58.4	0.018	0.65 (0.45–0.92)
Is there a smoker in your household?							
Yes	71/815	15/336	21.1/41.2	56/479	78.9/58.8	<0.001	0.36 (0.19–0.63)
No	128/1296	51/531	39.8/41.0	77/765	60.2/59.0	0.749	0.94 (0.64–1.36)

^a^ Adjusted for age and gender, standard logistic regression. ^b^ Adjusted for age and gender, Firth regression. For levels marked with “-”, the Firth regression model also failed to yield valid results. ^c^ Adjusted for gender, standard logistic regression. ^d^ Adjusted for age, standard logistic regression.

**Table 3:** Univariate analysis of PPV23 vaccination effectiveness.

Variable	No. of Vaccinated Individuals	Pneumonia (*n* = 66)		Non-Pneumonia (*n* = 867)		χ^2^ Value ^a^	*p*-Value ^a^
		*n*	%	*n*	%		
Survey locations						15.80	0.001
Shajiabang Town	228	29	12.7	199	87.3		
Changfu Street	122	9	7.4	113	92.6		
Zhitang Town	393	20	5.1	373	94.9		
Shanghu Town	190	8	4.2	182	95.8		
Age (Years)	933	66	100.0	867	100.0	22,215 ^c^	0.002 ^c^
Gender						14.26	<0.001
Male	421	45	10.7	376	89.3		
Female	512	21	4.1	491	95.9		
Education level						0.85 ^b^	0.819 ^b^
Primary school and below	829	58	7.0	771	93.0		
Middle School	96	8	8.3	88	91.7		
High school	7	0	0.0	7	100.0		
Associate degree and above	1	0	0.0	1	100.0		
Living status						1.84	0.399
Living alone	120	12	10.0	108	90.0		
Living with spouse	282	18	6.4	264	93.6		
Living with children	531	36	6.8	495	93.2		
Occupation before retirement						6.04 ^b^	0.512 ^b^
Teacher	3	0	0.0	3	100.0		
Healthcare worker	3	0	0.0	3	100.0		
Government employee	1	0	0.0	1	100.0		
Corporate employee	18	3	16.7	15	83.3		
Self-employed business owner	31	1	3.2	30	96.8		
Restaurant/Commercial service worker	9	1	11.1	8	88.9		
Worker/Migrant worker	143	8	5.6	135	94.4		
Farmer	648	50	7.7	598	92.3		
No fixed occupation	77	3	3.9	74	96.1		
Average monthly income (CNY)						3.21 ^b^	0.374 ^b^
<1000	575	37	6.4	538	93.6		
1000~2999	321	25	7.8	296	92.2		
3000~4999	29	4	13.8	25	86.2		
≥5000	8	0	0.0	8	100.0		
Medical insurance						0.07	0.785
Yes	817	59	7.2	758	92.8		
No	116	7	6.0	109	94.0		
Overall health status						2.64 ^b^	0.735 ^b^
Excellent	16	1	6.3	15	93.8		
Very good	208	13	6.3	195	93.8		
Good	323	27	8.4	296	91.6		
Fair	342	21	6.1	321	93.9		
Poor	38	4	10.5	34	89.5		
Don’t know	6	0	0.0	6	100.0		
Chronic disease						2.55	0.110
Yes	601	49	8.2	552	91.8		
No	332	17	5.1	315	94.9		
Chronic respiratory disease						25.65 ^b^	<0.001 ^b^
Yes	47	12	25.5	35	74.5		
No	886	54	6.1	832	93.9		
Awareness of pneumonia						0.44	0.509
Know	510	33	6.5	477	93.5		
Don’t know	423	33	7.8	390	92.2		
Do you consider pneumonia a serious disease?						0.95	0.620
Yes	339	27	8.2	312	94.8		
No	116	9	7.8	107	92.2		
Unclear	478	30	6.3	448	93.7		
Are you worried about getting pneumonia?						1.19 ^b^	0.747 ^b^
Very worried	42	3	7.1	39	92.9		
Somewhat worried	191	14	7.3	177	92.7		
Not worried	362	29	8.0	333	92.0		
Haven’t thought about it	338	20	5.9	318	94.1		
Do you think pneumonia can be prevented?						0.84 ^b^	0.416 ^b^
Can be prevented	497	38	7.6	459	92.4		
Cannot be prevented	9	1	11.1	8	88.9		
Unclear	427	27	6.3	400	93.7		
Have you heard of the pneumonia vaccine?						0.01	0.930
Yes	436	30	6.9	406	93.1		
No	497	36	7.2	461	92.8		
Do you know about the free pneumonia vaccine for the elderly?						0.97	0.324
Know	357	21	5.9	336	94.1		
Don’t know	576	45	7.8	531	92.2		
Would you be willing to receive the free pneumonia vaccine?						2.06	0.357
Willing	546	36	6.6	510	93.4		
Unwilling	115	6	5.2	109	94.8		
Unclear	272	24	8.8	248	91.2		
Have you ever received the influenza vaccine?						<0.01 ^b^	1.000 ^b^
Yes	29	2	6.9	27	93.1		
No	904	64	7.1	840	92.9		
Household location						5.93 ^b^	0.115 ^b^
Urban center	8	0	0.0	8	100.0		
Suburban area	30	3	10.0	27	90.0		
Rural town	210	22	10.5	188	89.5		
Rural dispersed	685	41	6.0	644	94.0		
Do you use air purifiers or ventilation systems at home or work?						0.79 ^b^	0.721 ^b^
Yes	32	1	3.1	31	96.9		
No	901	65	7.2	836	92.8		
Do you open windows for ventilation at home or work?						1.56 ^b^	0.212 ^b^
Yes	891	61	6.8	830	93.2		
No	42	5	11.9	37	88.1		
Hand washing frequency (times per day)						2.49 ^b^	0.320 ^b^
0	7	1	14.3	6	85.7		
1~3	183	17	9.3	166	90.7		
4~8	392	24	6.1	368	93.9		
>8	351	24	6.8	327	93.2		
Hand washing frequency with soap or hand sanitizer (times per day)						0.49	0.920
0	164	13	7.9	151	92.1		
1~3	453	33	7.3	420	92.7		
4~8	233	15	6.4	218	93.6		
>8	83	5	6.0	78	94.0		
Do you have a habit of washing hands before meals?						0.84 ^b^	0.307 ^b^
Yes	873	60	6.9	813	93.1		
No	60	6	10.0	54	90.0		
Do you have a habit of washing hands after returning home from outings?						0.23	0.634
Yes	776	53	6.8	723	93.2		
No	157	13	8.3	144	91.7		
Do you currently smoke?						2.26	0.133
Yes	194	19	9.8	175	90.2		
No	739	47	6.4	692	93.6		
Is there a smoker in your household?						6.05	0.014
Yes	351	15	4.3	336	95.7		
No	582	51	8.8	531	91.2		

^a^ two-sided χ^2^ test. ^b^ Fisher’s exact test. ^c^ Mann–Whitney U test.

**Table 4:** Multivariate logistic regression analysis of factors influencing PPV23 vaccine effectiveness.

Variable	Reference Group	β	$S_{\bar{x}}$	Wald χ^2^ Statistic	*p*-Value	OR (95% CI)
Survey location				15.81	0.001	
Changfu Street	Shajiabang Town	−0.44	0.42	1.10	0.293	0.65 (0.27–1.41)
Zhitang Town		−1.11	0.32	11.81	0.001	0.33 (0.17–0.62)
Shanghu Town		−1.27	0.43	8.95	0.003	0.28 (0.11–0.62)
Age (years)		0.07	0.03	5.45	0.020	1.07 (1.01–1.14)
Gender						
Female	Male	−0.87	0.29	9.23	0.002	0.42 (0.24–0.73)
Chronic respiratory disease						
No	Yes	−1.85	0.39	22.26	<0.001	0.16 (0.07–0.35)

## Data Availability

Data will be made available on request.
